# Backlash Against Globalisation and the Shadow of Phobos

**DOI:** 10.1007/s40647-020-00308-0

**Published:** 2020-11-09

**Authors:** Marek Rewizorski

**Affiliations:** Institute of Political Science, University of Gdańsk, ul. Jana Bażyńskiego 4, 80-309 Gdańsk, Poland

**Keywords:** International order, Economic globalisation, Fear, Populism, Hyperglobalisation

## Abstract

This article addresses challenges to the established international order. Its critique becomes particularly discernible at the domestic level, where political and economic frameworks are contested by electorates and anti-establishment movements in Western countries, engulfed by anti-globalisation sentiments and disillusioned with the asymmetric formula of globalisation. Populism implies that the ‘social revolt’ facing the West today, regardless of whether it takes place in France, Greece, Hungary, Poland, the UK or the USA, is not a legitimate response to deep-seated problems but rather is the problem itself. The main aims of this article are to investigate what has caused this surge of support for populism in the West; what the role of the default system of economic governance is in inspiring populist resentment; and, finally, what the identity crisis, stemming from ‘hyperglobalisation’, and wrecking the social order in Western societies has to do with fear, (un)fairness and the redistributive effects of economic globalisation.

## Introduction

In one of the *Worldviews* (2017) published by *The Washington Post*, an influential American political philosopher Francis Fukuyama expressed deep concern about the danger to the liberal order which comes from below the surface of international politics. Fukuyama, like many other observers (Eichengreen 2018; Finbow 2018; Linn 2017), is worried that the liberal movement across the West will soon fade away. In their view, an incoming political crisis signalled by the backlash of left and right-wing nationalisms on both sides of the Atlantic (Rodrik 2018), combined with grievances over immigration and multiculturalism (Greven 2016), will exacerbate internal tensions within democracies produced by globalisation, accelerate the erosion of political institutions, and dismantle democratic norms. Fukuyama (2018: 111) notes that the spectre of demagogic populism is revealed in “the common objective of populist politicians in both Europe and the United States that is to take back our country”. In his vision, the populist narrative is centred on a dyad of tenets: identity and resentment. Whilst the right-wing populists underscore the traditional understanding of national identity, diluted and misinterpreted by (un)cultural ‘others’ (migrants, newcomers, foreigners), the progressive left is contesting the very concept of identity as intolerant. The other tenet of the populist narrative–the politics of resentment–appeals to ‘the people’ who fear that their way of life will be destroyed, they will be deprived of dignity, and, finally, their religion or tradition will be disrespected. They are more susceptible to the narrative of populist leaders playing on emotions, fears, resentments or unspecified frustrations, which Panizza (2005) defines as “unmet demands”. Resentment is not only about the attribution of blame, but also about the demand for compensation of some kind from governments responsible for giving preferences, e.g. to business elites, bankers, or migrants who downsize the cost of labour.

Whilst resting on approach to populism as ideology, characterised by the moral and Manichean distinction between ‘the people’ and ‘the elite’, and the conviction that politics is about respecting sovereignty at any cost (Mény and Surel 2002; Mudde 2004; Mudde and Rovira Kaltwasser 2018), this article addresses the ‘populist revolt’ (Cuperus 2017; Cox 2017) and fear against globalisation, which is linked to economic anxiety and concerns about the destruction of traditional values by means of the modernisation of social relations endorsed by the globalisation-friendly elite. It will contribute to the literature explaining the eruption of populism by the downsides of globalisation and inconsistencies of global economic governance (Higgot 2018; Hoekman and Nelson 2018a, 2018b; Montier and Pilkington, 2017; Rodrik 2018), as well as the relationship between the emergence of populism and the occurrence of trade shocks and financial crises (cf. Autor et al. 2013, 2017; Dippel et al. 2018; Funke et al. 2016). By assuming that the populist discourse is not only a normal destabilising element within democratic politics and the political challenge of our age (Rosanvallon 2008), but a mainstream in the politics of contemporary Western democracies, named “the populist *Zeitgeist*” (Mudde 2004), this article will investigate what has caused this surge of support for populism in the West; what the role of the default system of economic governance is in inspiring populist resentment; and, finally, what the identity crisis, stemming from ‘hyperglobalisation’, and wrecking the social order in Western societies has to do with fear, (un)fairness and the redistributive effects of economic globalisation.

## What has Caused Surge of Support for Populism in the West?

The contestation of globalisation and the established international order by electorates in Western countries has been signalled predominantly by the building of support for populist movements, which dates back to 2010 and is expressed in their strengthened position in national elections (Durant et al. 2013). In the case of European elections, the surge of support for populists is even longer, having been sparked in 1984 by the right-wing National Front winning 11 per cent of the votes in the European elections in France (Ivaldi 2012). In 2019, right-wing populists in the West, despite various mindsets and practices, dominated the radical political scene. Cas Mudde (2019) in a commentary to the recent European Parliament’s (EP) election observed three emerging trends. The first is characterised by “increased fragmentation, growing support for populist parties, and the decline of the center-right and center-left blocs” (Mudde 2019: 23). The second is the big losses of left-wing populists such as Syriza or Podemos compared to the 2014 EP elections, and non-radical populism losing momentum (The Five Star, The Finns Party, Bulgaria Without Censorship). The third one, signalling the viral nature of right-wing populism, is the transformation of erstwhile conservative governing parties in Central Europe–Fidesz in Hungary and PiS in Poland–into a “populist radical right in the wake of the so-called ‘refugee crisis’ of 2015, and the Jihadist terrorist attacks in Brussels and Paris around that time” (Mudde 2019: 24) and thus swinging the voting power in Brussels towards more radical-oriented mindsets. As noted by Ruth Wodak (2015: 7), right-wing populism, which she defines as “a political ideology that rejects existing political consensus and usually combines laissez-faire liberalism and anti-elitism”, has a set of four common dimensions which are salient for all parties in this group. These include nativist/ethnonationalism, anti-elitism, authoritarianism and ‘law-and order-politics,’ and conservative values in some specific context, accompanied by strong leadership, chauvinism, revisionism, nativism, anti-intellectualism, de-historisation and homeland rhetoric (Cohen-Almagor 2017). These features fill the spectrum between the two extremes outlined in the minimalistic definition of populism (Mudde 2004: 543) as “an ideology that considers society to be ultimately separated into two homogeneous and antagonistic groups, ‘the pure people’ versus ‘the corrupt elite’, and which argues that politics should be an expression of the volonté générale (general will) of the people”. As such, populism, representing the Manichean division between the ‘forces of pluralism’ and ‘forces of elitism’ and a regular feature of politics in Western democracies since the 1980s, not only maintains a problematic relationship with democracy but is also becoming one of the main challenges to the liberal democratic regime (Canovan 1999, 2005). Reflecting on populists’ claims to be ‘true democrats’, Marget Canovan (1999: 7) observed that populism is not anti-democratic, as it rests on “mobilizing the electorate against established power-holders and opinion-formers”. It is rather directed against liberal democracy, which influential American political theorist Robert Dahl (1971: 2) named a “polyarchy” and defined as “a political system one of the characteristics of which is the quality of being completely or almost completely responsive to all its citizens”.

For Canovan (1999) populism stems from tensions between two opposing styles of modern politics, dubbed as “two faces of politics”. The first–one the pragmatic face–looks towards polyarchy. Criticised as an elite theory of democracy (Krouse 1982; Skinner 1973), it underscores elitist support for traditional institutions and practices such as “multi-party systems, free elections, pressure groups, lobbying and the rest of the elaborate battery of institutions and practices by which we distinguish democratic from other modern polities” (Canovan 1999: 11). The second face of democracy, which Canovan describes as redemptive, is centred on “the promise of a better world through action by the sovereign people (…) a strong anti-institutional impulse: the romantic impulse to directness, spontaneity and the overcoming of alienation” (Canovan 1999: 11). The tensions between the pragmatic and redemptive faces of democracy that are exacerbated by the situation in which power is more a result of political adjustments than the will of the people lead to the redemptive failure of a better world through action by the sovereign people, which creates a perfect breeding ground for populist movements. The latter seek to replace corrupt or fraudulent elites, to create an opportunity for reducing the distance between the people and government, and making their will heard more. The mechanism that has caused this surge of support for populism (or illiberal democracy, as suggested by Fareed Zakaria, cf. Zakaria 1997) in the West is therefore based on the oppositions and tensions between ‘the elites’ as pragmatic forces supporting liberal institutions and practices and ‘the people’ unable to express their general will. The by-product of tensions–populists who promise better life for those left behind by decision- and opinion-makers, disenfranchised by economic globalisation and disillusioned with liberal solutions proposed by the leaders of political parties–may have different objectives. As noted by Cas Mudde and Cristóbal Rovira Kaltwasser (2012), populism with a strong anti-elitist angle can lead to the marginalisation of specific groups of society, the weakening of political institutions, culminating in the undermining of minority rights and protections. In a similar vein, Dani Rodrik (2018) suggests that the populist backlash against political elites and globalisation can intensify the formation of new political cleavages, deter or prevent compromises, or undermine the legitimacy or power of political institutions. In his view, populists may target liberal democracy and the established international order in two ways. One is that populist leaders can mobilise ‘the people’ along ethno-national/cultural cleavages when the globalisation shock becomes salient in the form of immigration and refugees (Rodrik 2018: 2). This shock effect is exemplified by the political practice in Poland and Hungary, where erstwhile conservative mainstream political parties PiS and Fidesz have targeted liberal, non-traditional social values as well as ‘delocalized’ elites (judges, journalist, academics), considered as the vanguard of the pro-globalisation forces that are detached from the homeland rhetoric (cf. Krekó et al. 2018). The second way of playing on resentment is to mobilise supporters along income/social class borders, where the globalisation shock is rooted in the transformation of international trade and finance, and becomes apparent to the poorer social strata of Western countries (Pierce and Schott 2012; Autor et al. 2013). Indeed, there is a widespread view that the effects of globalisation, including unfair international trade, capital mobility, and recurring economic and financial crises, may lead to increased unemployment, reduced wages, stalling growth rates and deprivation, which stirs dissatisfaction and radicalisation (Funke et al. 2016). Nevertheless, the question of the specific role of the economic factors of populism, remains open despite rich empirical evidence. Not only do the economic explanatory variables focused on poor economic performance and economic deprivation compete for attention in the academic discourse with a conservative-nostalgic backlash against progressive value change and loss of cultural identity (cf. Inglehart and Norris 2016), but they are also analysed from diverse theoretical perspectives (comparative political economy, modernisation theory, structuralism and post-structuralism, political psychology and democratic theory), and methodological approaches (discourse analysis, archival research, and formal modelling) (see Gidron and Bonikowski 2013). Finally, the economic variables of populism are criticised as mixed and inconsistent. Supporters of this view indicate that in some developed Western countries, such as Italy and Spain, whose economies have suffered gravely from the downsides of globalisation, the economic crisis, and inconsistencies in regional economic governance (Eurozone), populist pressure is experienced less severely than in other Eurozone countries such as Austria or the Netherlands, economically well-off and relatively untouched by the recent downturn in prosperity (Springford and Tilford 2017). Going further, in Central Europe, considered as the Eastern frontier of the West, inequality and socio-economic deprivation, whilst definitely creating fertile grounds for the rise of authoritarian populism, fail to explain its political success. The right-wing populist trend is not an economic populism, it rather targets identity-based fears and nationalist sentiments (Stępińska et al. 2016). The shortcomings of the economic factors explaining the rise of populism demand a more nuanced politico-economic approach, suitable in an analysis of how economic globalisation is, or may be used by populist parties in the West.

## Globalisation, Economy, and Populism

Dani Rodrik (2018), in a similar way to Cas Mudde, builds the idea of populism upon the concept of society splitting into two antagonistic camps pitted against one another. In his definition, populism means “an anti-establishment orientation, a claim to speak for the people against the elites, opposition to liberal economics and globalization, and often (but not always) a penchant for authoritarian governance”. Considered as a form of political thinking which refers to social fears and resentment, and pertains to such social movements whose leaders seek popularity amongst the public in order to manipulate people and lead them towards nebulous, albeit attractively formulated goals, populism is widespread in many countries at different levels of socio-economic development. As a ‘thin’ ideology (Mudde 2004), populism is characterised by such markers as the criticism of closer international political integration (the United Kingdom), opposition to the regulations of the economic and monetary union of the EU (Greece, Spain and Portugal), a programmatic fight against the pro-globalisation elite and a negative attitude to migrants, who are considered a threat to national security (Hungary and Poland), anti-trade nativism (the United States), and economic nationalism (numerous Latin American countries), to name just a few.

The economic sources of populist resentment and their opposition to liberal economics and globalisation are discernible in specific populist agendas targeting globalisation as a ‘thick’ ideology weakening nation-states, undermining sovereignty and democracy, eroding international cooperation regulatory mechanisms and misleading ‘the people’ with the empty promises of the elitist framework of global economic governance (Higgot 2018). Dani Rodrik, known for his critical approach to deeper economic integration (cf. Rodrik 2011, 2017) calls global governance a “false promise” (Rodrik 2017: 206) arguing that civil society is national, and so are nation–states, and the world economy should be cured at the domestic level, where most of the failures of global trade and finance are born (Hoekman and Nelson 2018a). Whilst seeking the major drivers of the rise of populism in the uncompensated adjustment costs and redistributive effects of economic globalisation, Rodrik fleshes out the matter of inequalities and sovereignty being restricted by the ‘dark spectre’ of globalisation, represented by the transnational bureaucratic and business elite, operating above heads of states. His reflexion is reminiscent of the ideas of the school of critical theory in International Relations (cf. Moolakkattu 2009). They focused on the emergence of hubs of governance which combine inter- and transnational regulations, public–private regimes, the forms of private governance and self-regulation, constituting a new form of global polity (Cox 1994; Ougaard 1999). Robert Cox named these new structures “global nebula”. A similar view was expressed by Susan Strange, the *grande dame* of international political economy, who observed that both formal and informal entities which enjoy a considerable scope of autonomy, and have assets at their disposal can play the role of originators of ideas. Such ideas are subsequently propagated as mental constructions by the highest echelons of state officers, executives in international businesses, specialists and experts sitting on working groups, meeting at conferences and forming epistemic communities (Strange 1996: 62).

Both Robert W. Cox and Dani Rodrik in their broad views on economic globalisation accurately point out the risk of pushing globalisation to socially unacceptable limits in developed societies, dubbed “hyperliberalism” (Cox and Sinclair 1996) or “hyperglobalization” (Rodrik 2011). Cox (1996) looks at hyperliberalism as the fundamental transformation of the neoliberal form of state into its excessive, hyperliberal version, materalised as an effect of denouncing the contract worked out with capital and labour during the post-war economic boom. Prepared and introduced by the collective effort of the government-business or “global nebula” alliance, it rules out corporate-type solutions like negotiated wages and price policies, and relegates employment and welfare commitments to a lower league. The restructuring of production leads to accentuating segmentation and division within the working class (e.g. between migrant workers and local established workers), and juxtaposing the ‘haves’ and ‘have-nots’. It also greatly expands the list of disadvantaged and excluded groups experiencing hardships, from the unemployed, as direct welfare recipients, through farmers and small businesses to workers in sensitive industries, such as textiles, automobiles, steel or shipbuilding, all thrust into precarious conditions due to reduced real wages and temporary contracts. Populists master the use of the capital of social frustration that has been accumulated over many years, and politically mobilise a disenfranchised majority against a small majority (or the elite) and their hyperliberalist policy orthodoxy (Cox 1996: 191–208). This opposition between excluded and included groups of society, and the process of storing up the capital of resentment and social anger is discernible in Western societies along income/social class lines, as illustrated by American political scientist Ian Bremmer (2018) in one of his studies. He depicts a situation in which the process of economic liberalisation in the United States contributes to the growth in GDP and average income of national economies whilst leading to an uneven distribution of benefits. As the differences in affluence increase, this distribution is viewed as more and more unfair. Bremmer emphasises that this process is spread over time, saying that.

The incomes for the bottom half of earners in the United States remained flat between 1980 and 2014, while the income for the top 0.001 percent of the richest Americans surged a jaw-dropping 636 percent. The top 1 percent of U.S. adults earned 27 times what the bottom 50 percent earned in 1980. By 2016, it was 81 times higher.

(Bremmer 2018: 27).

If we add the recurring subject of high compensation for bankers (who are thus rewarded for mismanagement), unclear remuneration systems in banks and state enterprises (which circumvent the regulations on excessive salaries), the unbridled desire of representatives of the political class to increase their remuneration and bonuses, which are over ten times or so higher than median salaries, it comes as no surprise that anti-establishment movements relish making the issues of the distribution of wealth the primary element of their election campaigns (King 2017).

Twenty years after Robert Cox indicated hyperliberalism by as ‘a would-be’ new policy orthodoxy, Dani Rodrik draws a picture of hyperglobalisation as a real “agenda of economic liberalization and deep integration” (Rodrik 2011: xvii), and the post-war globalisation model beyond its limits, where.

[t]rade agreements [were] extended beyond their traditional focus on import restrictions and impinged on domestic policies; controls on international capital markets were removed; and developing nations came under severe pressure to open their markets to foreign trade and investment. In effect, economic globalization became an end in itself.

(Rodrik 2011: xvii).

Observing the incompatibility of this ultimate form of economic globalisation with democracies which have the right to protect their social arrangements, Rodrik (2018: 38) advises “rebalancing in three areas in particular: from capital and business to labor and the rest of society, from global governance to national governance, and from areas where overall economic gains are small to where they are large”. However, the fundamental problem with the ‘Rodrikian’ solution to the populist challenge is that reconstituting the international economy with a stronger emphasis on sovereignty may open the door for pursuing distinct national policies which not only overlap with the economic right-wing populism, but dismantle the benefits of international institutional cooperation obtained so far (Hoekman and Nelson 2018b). Giving too much policy space to governments which are free from constraints imposed by international institutions (such as trade agreements or international regimes) and free to adopt policies allowing national economic development goals to be achieved can also alter the calculus of risk and support for the key agents of structural change in global economic governance (states, non-state actors, corporations and individuals such as Donald Trump, Valdimir Putin or Xi Jinping) (cf. Wright 2017). Thus it may amplify anti-globalisation signals and boost rolling back liberalisation, and increase discrimination against foreigners migrants, companies, or investments perceived as a threat to national security. What is more, non-restricted national governance may work as an amplifier of external threats, such as trade shocks, economic and financial crises, or a justification of rolling back compensation mechanisms (social welfare/safety nets) as a result of pursuing austerity policies.

Whilst Rodrik makes a good point in seeing major drivers of the rise of populism in the uncompensated adjustment costs and redistributive effects of economic globalisation, his deep criticism of hyperglobalisation and call for a decisive shift towards national governance does not guarantee avoiding the extreme unfairnesses of globalisation, and playing on fears of its consequences by populists. The middle way between hyperliberalism/hyperglobalisation and a more national ‘policy space’ approach, may rather be improving the quality of domestic policies by identifying good regulatory practices and encouraging learning which, in turn, might decrease the burden placed on domestic policy, enhance good governance (predominantly in the domain of evidence-based public policy), and influence perceptions of globalisation as a (more) fair process, instead of pointing to it as the cause of the current malaise. Obtaining these goals is dependent on developing international cooperation in spheres of global governance which go far beyond foreign trade and investment, which Rodrik considers as the main external factors affecting the area of national policy.

One example here is the global value chain revolution, which started at the end of the twentieth century, and had visibly reshaped the division of power in the world. Richard Baldwin called it the “second unbundling”, as ICT made it possible to offshore know-how at almost no cost from the most developed countries of G7 towards developing countries (Baldwin 2016). Technology flow was defined by international production networks, rather than simply national borders. Countries which combined a cheap labour force with cheap access to know-how have benefited the most. This new globalisation was transformative, revolutionary and disruptive in many areas, and within a short period of time produced ‘the great convergence’.

An even more exemplary case of international cooperation which translates into the opportunity to reduce the burden placed on domestic policy is the G20 plan to close the global infrastructure gap. The infrastructure investment gap can be defined as the difference between a country’s investment needs, and what is likely to be spent under current trends. According to data published in the Global Infrastructure Outlook in July 2017 encompassing 50 countries in 7 sectors (energy, telecommunication, transports, airports, railways, roads and ports, and water), global infrastructure investment needs to be as high as $94 trillion between 2016 and 2040. To meet this investment need, the world will have to increase the proportion of GDP it dedicates to infrastructure to 3.5 per cent, compared to the 3.0 per cent which is spent now (Global Infrastructure Hub 2017). International cooperation in filling a financing gap identified in infrastructure in advanced economies has become crucial at least for two reasons. One is that most of the post-World War II era infrastructure assets are approaching the end of their useful lives and need replacement. At the same time, government budgets and, consequently, infrastructure supply are increasingly constrained as a result of the global financial crisis, and more recently the COVID-19 pandemic pushing economies into a Great Lockdown, which helped contain the virus and save lives, but also triggered the worst recession since the Great Depression. The second reason is that investing in infrastructure gives opportunities to create a pool of jobs, leads to a quicker resumption of economic activity, and eases the effects of the above-mentioned hyperliberalist policy orthodoxy underscored by Robert Cox. Disarming the bomb of resentment and social anger discernible in Western societies may be possible thanks to the efforts of the G20, which between 1998 and 2008 became the apex forum burdened with the task of tailoring an appropriate mechanism of global governance and responding to the various needs that had been raised by members of the international community (see Cooper and Thakur 2013). In 2018, the Argentinian G20 presidency introduced a plan to fill the perceived financing gap in infrastructure (G20 2018). The three pillars of the projected solution, namely (1) using public finance (e.g. taxes, pensions, user fees for infrastructure services, and guarantees) to leverage or catalyse private sector investment, particularly long-term institutional investment; (2) strong commitment to build pipelines of ‘bankable’ projects, with an emphasis on megaprojects which are financed and operated through public private partnerships (PPPs); and (3) improving mechanisms to quickly replicate PPPs, could contribute to diminishing the burden placed on domestic policy, and by recognising the value of deeper international cooperation could be a part of response to the drivers of populism.

## Shadow of Phobos

Finding alternative path towards globalisation based on international cooperation and greater regulation may be useful as recent surveys on the populist vote in Europe and the perception of knowledge and policy preferences of European citizens towards globalisation and European integration (de Vries and Hoffmann 2016, 2018) show that arguments for reconstituting the international economy and politics towards more sovereignty-oriented solutions can be used by populists who are aware of the Western societies’ anxieties and fears of globalisation as an elite project. The findings in the first survey show that the fear of globalisation and economic anxiety is the decisive factor behind demands for changes to be implemented by the political mainstream (de Vries and Hoffmann 2016). The anxiety level amongst populist parties’ voters is inversely proportionate to their level of education, disposable income, and age (generally older people are more prone to see globalisation as a threat). Moreover, those who feel close to populist (particularly right-wing) parties are mainly motivated by fear and economic anxiety, although considerable differences by country are observed (see Table 1). Whilst in Austria and France a majority of respondents perceive globalisation as a threat (55 and 54 per cent, respectively), French and Italian respondents display the highest level of economic anxiety at 51 and 45 per cent respectively. In the UK, the number of people who feel economically anxious and see globalisation as a threat is particularly low, at only 26 per cent (cf. de Vries and Hoffmann 2016: 12–14). The opinions of UK respondents look surprising, given the results of the Brexit vote and politico-economic perturbations regarding the final version of the divorce agreement with the European Union.

**Table 1:** Key concerns for supporters of right and left-wing populist parties in the EU. *Source*: de Vries and Hoffmann 2016.

Right/Left Wing Populist Parties Supporters	Key concerns		
Right Wing	Globalisation as a threat (%)	Economic anxiety (%)	Traditionalism (%)
AfD (Germany)	78	49	46
FN (France)	76	67	67
FPÖ (Austria)	69	52	47
LN (Italy)	66	54	55
Fidesz (Hungary)	61	26	44
PiS (Poland)	58	20	67
PVV (Netherlands)	57	37	63
UkiP (GB)	50	32	53

Left Wing	Globalisation as a threat (%)	Economic anxiety (%)	Traditionalism (%)
Front de Gauche (France)	58	53	50
Die Linke (Germany)	54	44	28
MVCS (Italy)	48	52	49
MSZP (Hungary)	45	32	46
SP (Netherlands)	45	40	49
PODEMOS (Spain)	43	49	43

Economic anxiety is less intensive in Poland and Hungary, where the main right-wing populist parties PiS and Fidesz target identity-based fears and nationalist sentiments (traditionalism), using the concept of the nation, and not that of class (Krekó et al. 2018). The main concern for both leaders Jarosław Kaczyński and Victor Orbán is expressed in a belief that the *multi-kulti* West is imposing Muslim refugees on their countries in order to destroy their national identities (Balcer 2017). Kaczyński has made the following remark.

Today Poland is subject to pressures regarding the shape of our life, the situation of the average Pole; the shape of our society. We are being persuaded to radically change, to create a multicultural society, to create a new identity. (…) This is a matter of sovereignty. If we maintain it, we will defend ourselves. (…) If there is no strong identity, society can be made to do anything

(wPolityce 2016).

Defending traditional values and embarking on a mission “to carry those values to Western Europe and to defend those values against all attacks” (TVN24 2016) as a leitmotif of populist parties on the Eastern frontier of the EU, does not change the people’s knowledge and feelings about globalisation. A survey on globalisation and European integration (de Vries and Hoffmann 2018) has shown that considerable numbers of Europeans think that globalisation is a threat (44 per cent EU-wide, with national results varying from 53 per cent in Poland to 39 per cent in Spain), even though their personal experiences with globalisation have overall been more often good than bad. Globalisation can be best explained to Europeans in the economic context as the growing interconnectedness associated with trade (increased movement of products for 19 per cent of respondents and money for 17 per cent) and migration (increased movement of people for 17 per cent of respondents). (Table 2)

**Table 2:** Meaning of *globalisation* in five major European countries. Globalisation is the movement of… *Source*: de Vries and Hoffmann 2018.

Country	Products	Money	People	Technology	Culture
France	21	18	17	14	11
Germany	18	18	16	13	14
Italy	18	16	17	13	12
Poland	19	15	19	13	11
Spain	19	19	16	14	11

However, the proportion of those who feel that globalisation is a threat rather than an opportunity varies amongst EU societies (Table 3).

**Table 3:** Globalisation as a threat or opportunity in the five major EU member states. *Source*: de Vries and Hoffmann 2018.

EU member states	Opportunity	Threat
EU27	56	44
France	49	51
Germany	57	43
Italy	57	43
Poland	47	53
Spain	61	39

The results presented in tables 1–3 allow a set of conclusions to be drawn. Firstly, the fear of globalisation is expressed mainly by the supporters of right-wing populist parties. Secondly, for Europeans, globalisation goes hand in hand with the economy (globalisation as the movement of products, money and people, whilst the movement of technology and culture is indicated less often). Thirdly, a significant number of Europeans think that globalisation is a threat (44 per cent). Fourthly, given the span of fear amongst European societies (from 39 per cent in Spain to 53 per cent in Poland) there is a considerable reservoir of real/potential votes for right-wing anti-European and anti-globalisation parties. Fifthly, right-wing populists can use the power of resentment to attack globalisation in three ways. One is unfair trade and trade shocks as a source of economic anxiety magnified by movements of imported products hurting domestic business (Autor et al. 2013, 2017; Dippel et al. 2018; Rodrik 2018). The second one involves deregulated money and finance, portrayed as sources of economic and financial crises which undermine trust in markets, state institutions, and increase economic and political insecurity (cf. Funke et al. 2016; Guiso et al. 2017). Finally, there is immigration, depicted as a harbinger of adverse labour market effects, especially for less-skilled people, which populist parties take advantage of by blaming the elites for the crises and promising short-term protection (cf. Halla et al. 2017; Becker and Fetzer 2016).

## Unfair Globalisation and Extreme Crises

The contestation of political and economic mechanisms is the foundation of the anti-establishment parties both on the political right (e.g. UKIP in the United Kingdom and AfD in Germany) and on the left (e.g. the Five Star Movement in Italy, Syriza in Greece and Podemos in Spain). They use their respective electorates’ dissatisfaction with the pro-liberalisation and pro-globalisation elites, that divide wealth and influence amongst themselves in a way that is unfair and socially unacceptable (Rodrik 2018). The backlash against ‘unfair’ globalisation is underscored in numerous empirical studies which analyse the factors behind this rise. Their results stress that economic factors such as trade shocks and economic/financial crises play a crucial role in the rise of populist parties and support from voters. A study by Hicks and Devtaj (2017) can serve as the point of departure in tracing the links between the effects of trade globalisation and the frequently dramatic transformations in national labour markets. Hicks and Devtaj estimated that 13 per cent of jobs in the North American industrial sector were liquidated in 2000–2010 due to increased imports. Autor, Dorn and Hanson (2016) attributed a 10 per cent drop in employment in US industrial plants in 1991–2011 to the ‘import shock’ triggered by excessive competition from imports from China. They estimated that, over the period in question, import shock directly caused the liquidation of 2–2.4 million jobs in US industrial plants. Additionally, Autor et al. (2013; 2016; 2017) underscored the importance of the above-mentioned ‘import’ or ‘China shock’ that stemmed from Beijing acceding the WTO, which has led to higher unemployment, lower wages, and more governmental transfers in Western countries. In a recent study Autor, Dorn, Hanson and Majlesi (2017) examined the exposure of local labour markets to increased import competition from China. Not only did they find a positive correlation between rising import competition and Republican vote share gains, but also confirmed earlier research on the negative impact of import competition on employment and earnings in trade-exposed local labour markets (Autor, Dorn, and Hanson 2013) and a decreased likelihood that moderate politicians will win congressional elections (Autor, Dorn, Hanson, and Majlesi 2016). The results of their research lead to the conclusion that people react to economic upheavals by voting for more polarising candidates in US elections, and supporting the formation of extreme right-wing movements (e.g. Tea Party). Decisions of voters nurture populism which, as noted by Paul Taggart (2004: 275), “is a reaction to a sense of extreme crisis” that “spills over into a critique of politics and into the sense that politics as usual cannot deal with the unusual conditions of crisis”. Recent studies conducted in Germany (Dippel et al. 2018) revealed a similar effect of import exposure on voting for the extreme right, which is explained by (mediated by) the effect of import exposure on labour markets. What is more, it confirmed the evidence provided in the two surveys focused on the fear of globalisation analysed earlier. Amongst the supporters of the political left and right in Germany, the opposition against globalisation has been recorded predominantly on the right and far right in the last two decades (cf. Dippel et al. 2018; Mughan et al. 2003). Contrary to the political left, which criticises the unfair and profit-oriented world order, the far right rejects globalisation per se as the global spread of the capitalist economic system ruled by big money, and keeps strictly to the national framework (cf. Sommer 2008).

Interestingly, the adverse impact of trade globalisation on employment prospects is the *leitmotiv* of both left and right-wing populists who, at the same time, typically do not campaign against technological transformation and workers being replaced by automated manufacturing. Instead, their narrative refers to disregarded needs, and neglected demands for the benefits that result, in their view, from an unfair global trading system. Such voices strongly resonate with the public when populist leaders give the reasons for the lack of economic security, falling income and unemployment, and pointing to who should be blamed. One of the main ‘perpetrators’ is unfair international trade, confined within a global system shaped by rivalry between the values, ideas and material interests of the electorate on the one hand and the preferences of lobbying groups, who have access to decision-makers with a direct influence on wealth distribution, on the other. Helen Milner (1997) painted a highly intuitive, yet accurate, picture of this division. She noted that.

cooperation among nations is affected less by fears of other countries’ relative gains or cheating than it is by the domestic distributional consequences of cooperative endeavours. Cooperative agreements create winners and losers domestically; therefore they generate supporters and opponents

(Milner 1997: 9).

The opponents of cooperative agreements who join the ranks of populist movements point to international trade as a politically sensitive issue. As a result, they choose international trade as the target of their attacks. This is the mechanism of finding a ‘scapegoat’, which is employed with delight by populist leaders who readily blame all economic mishaps on foreigners, the Chinese who generate the import shock, Germans who export unemployment to neighbouring countries where they establish their assembly plants and chain stores, or Mexicans who take jobs away from Americans under the NAFTA agreement.

Not only trade shocks, but also economic and financial crises are used by populists to convince voters to turn to protectionist, populist, and nationalist policy agendas. Evidence analysed by Funke, Schularick, and Trebesch (2016), who focused on the political aftermath of financial crises from historical perspective, covering 20 countries and spanning 140 years with more than 800 elections between 1870 and 2014, shed light on three significant findings. One is that the financial crises are followed by important changes in voter behaviour that, in turn, contribute to high levels of political uncertainty. The latter is concomitant with the political polarisation which was exacerbated after financial crises throughout the nineteenth and twentieth centuries. Far right parties profit more from the financial and economic systemic default than the far left, in the short and long term. During the first five-year period after a crisis, right-wing populists increase their voting shares on average by about 30 per cent relative to their pre-crisis levels. In the longer run, they establish a solid base of voters lured by a political agenda responding to nationalistic tendencies. The second finding connects the radicalisation of voters with difficulties with governance, which becomes much more than a technical exercise after financial crises. In the aftermath of financial crises “government majorities tend to shrink, while parliamentary fractionalization rises” (Funke et al. 2016: 235–240). What is more, nowadays, the political fragmentation leading to government crises and changes in the executive branch is more intensive in comparison to political downturns observed in the 1980s. Political uncertainty, and increasing difficulties in governing are linked to the third, and probably the most troubling finding, namely to rising social unrest in the form of general strikes, violent riots and anti-government demonstrations which may be seen as an additional proxy for political constraints on governing. Harold R. Kerbo (1982) dubbed such anti-austerity protests as “movements of crisis”. Their emotional engines were, and still are focused on the opposition to hiperliberalism/hyperglobalisation (breaching the social contract between the elite and the people) and rolling back social security, resulting in cuts in public expenditure, unemployment and rising inequality. The recent wave of the movements of crisis spilled over several Eurozone countries such as Italy, Portugal and Spain in Southern Europe, as well as Ireland and the UK. However, the anti-austerity unrest in Greece in the aftermath of the sovereign debt crisis in the Eurozone that accelerated in 2010, was much more intense than elsewhere. As noted by Rüdig and Karyotis, it was to some extent a crisis of Greeks who had been disenfranchised by economic globalisation and “perceived themselves as facing the toughest economic conditions, with more than 60 per cent considering their economic positions to be ‘rather bad’ or ‘very bad’” (Rüdig and Karyotis 2014: 492). Also in this case, an extreme crisis and the unresponsiveness of the political elites has spread feelings of dissatisfaction amongst people.

## Conclusion

The political and economic analysis in this article focused on sources of populist resentment and their opposition to liberal economics and globalisation, discernible in specific populist agendas targeting globalisation as a ‘thick’ ideology, weakening nation-states, undermining sovereignty and democracy, eroding international cooperation regulatory mechanisms and misleading ‘the people’ with empty promises of an elitist framework of global (economic) governance. The latter has come under heavy fire, indicating the incompatibility of hyperliberalism/hyperglobalisation, as the ultimate form of economic globalisation, with democratic countries, which have the right to protect their social arrangements. Dani Rodrik is amongst the critics arguing that globalisation and global economic governance be recast along national, democratic, and sovereign lines. However, the ‘Rodrikian solution’ is challenging for both domestic politics and the established international order. Reconstituting the international economy with a stronger emphasis on sovereignty may open the door to pursuing distinctive national policies, which not only overlap with economic right-wing populism, but dismantle the benefits of international institutional cooperation. If globalisation cannot be abandoned and the alternative of recasting politics and economics along national, democratic, and sovereign lines is arguable, perhaps the most feasible solution to the current malaise would be to choose a middle way between hyperliberalism/hyperglobalisation and a more national ‘policy space’ approach. This might be expressed by improving the quality of domestic policies, by identifying good regulatory practices and encouraging learning which, in turn, might diminish the burden placed on domestic policy, enhance good governance (predominantly in the domain of evidence-based public policy), and influence perceptions of globalisation as a (more) fair process, instead of pointing to it as the cause of the current malaise. Obtaining these goals is dependent on developing international cooperation in the spheres of global governance which go far beyond foreign trade and investment, which Rodrik considers as the main external factors affecting the national policy space.

Finding an alternative path towards globalisation based on international cooperation and greater regulation may be useful, as recent public opinion surveys indicate that a considerable part of Western societies continue to express their fear of globalisation, which is linked to economic anxiety and concerns about the destruction of traditional values by means of the modernisation of social relations endorsed by the globalisation-friendly elite. This fear of economic globalisation, personified in this article as Phobos, son of Ares and Aphrodite, is expressed mainly by the supporters of right-wing populist parties. Right-wing populists use the power of resentment and attack globalisation in three ways. One is unfair trade and trade shocks, the second is economic and financial crises and the third one migration, depicted as a harbinger of adverse labour market effects for less skilled people. Interestingly, despite referring to the wrongdoings of globalisation, right-wing populists confine themselves to blaming the elites for the crises and promising short-term protection. At the same time, they express scant political support for more redistribution. Recent research (cf. Pikkety 2018) partly explains this unwillingness to act by the multidimensional political conflict in the Western political systems generating deep social cleavages, and the pro- and anti-globalisation divides of those who are in favour of redistribution. These divides translate into extreme difficulties in coalition-building in favour of redistribution, not only on the right but also on the political left. This is, however, only a partial explanation. The effective and comparable distribution of both economic and non-economic benefits becomes even more complex, given the role of external factors, the influence of which is more observable in the historical perspective of the last two centuries. Governments that disregard them can generate long periods of political fragmentation and set the stage for a new wave of movements of crisis that vehemently reject the benefits of globalisation.
